# Supplementary material for: Suppression of RBFox2 by Multiple MiRNAs in Pressure Overload-Induced Heart Failure
Source: Int J Mol Sci. 2023 Jan 9;24(2):1283. doi: 10.3390/ijms24021283 (PMC9867119; doi:10.3390/ijms24021283)
Supplement: Supplementary file 1 [file ijms-24-01283-s001.zip › Supplementary Figure-20221224.pptx]

## Slide 1
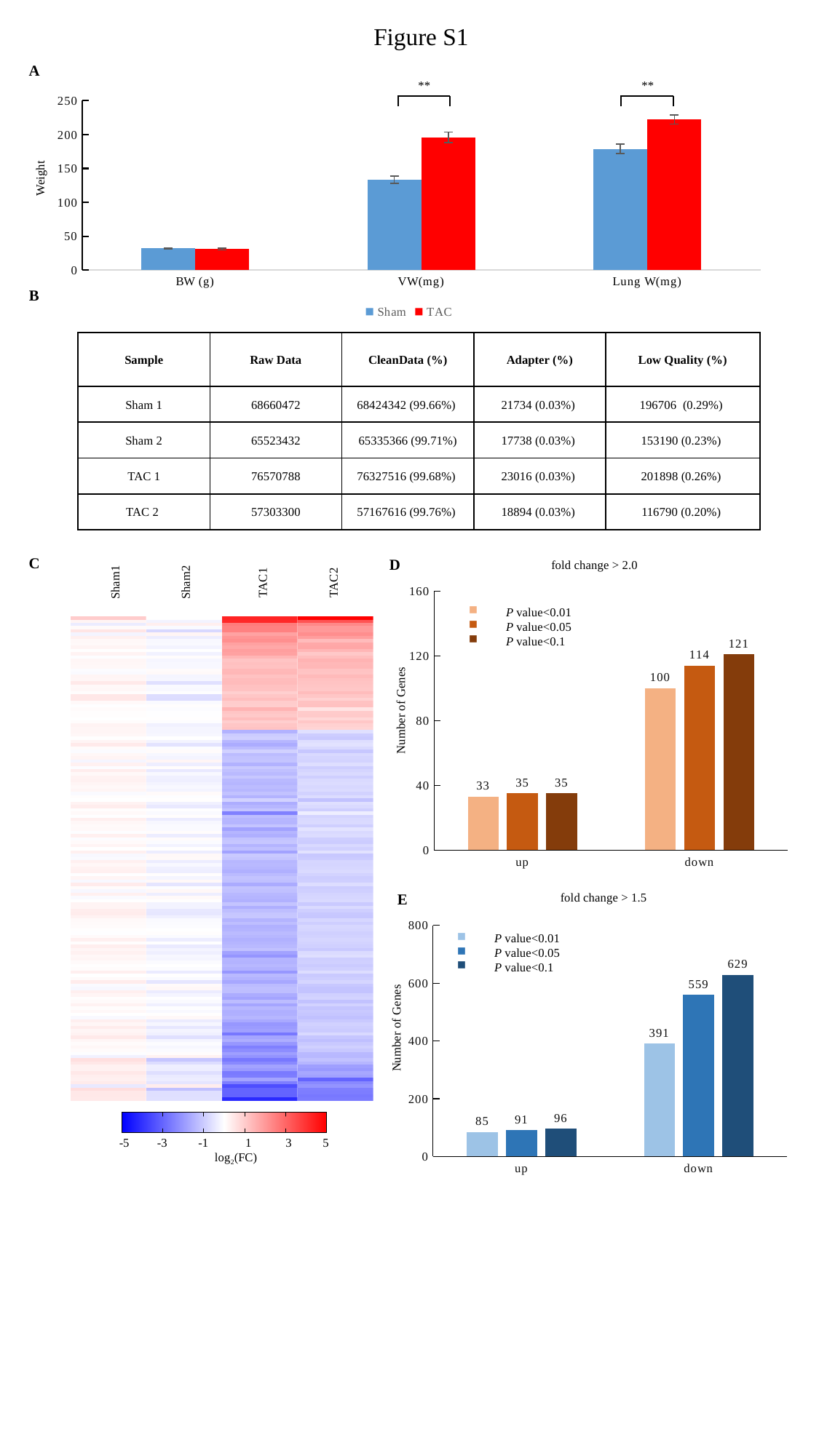

Figure S1
A
**
**
### Chart
| Category | Sham | TAC |
|---|---|---|
| BW (g) | 32.2333333333333 | 30.966666666666665 |
| VW(mg) | 133.58333333333334 | 195.6833333333333 |
| Lung W(mg) | 179.03333333333333 | 222.08333333333334 |Weight
B
| Sample | Raw Data | CleanData (%) | Adapter (%) | Low Quality (%) |
| --- | --- | --- | --- | --- |
| Sham 1 | 68660472 | 68424342 (99.66%) | 21734 (0.03%) | 196706 (0.29%) |
| Sham 2 | 65523432 | 65335366 (99.71%) | 17738 (0.03%) | 153190 (0.23%) |
| TAC 1 | 76570788 | 76327516 (99.68%) | 23016 (0.03%) | 201898 (0.26%) |
| TAC 2 | 57303300 | 57167616 (99.76%) | 18894 (0.03%) | 116790 (0.20%) |
C
D
fold change > 2.0
Sham1
Sham2
TAC1
TAC2
-5 -3 -1 1 3 5
log2(FC)
### Chart
| Category | FPKM>1 | FPKM>1 | FPKM>1 |
|---|---|---|---|
| up | 33.0 | 35.0 | 35.0 |
| down | 100.0 | 114.0 | 121.0 | P value<0.01
 P value<0.05
 P value<0.1
Number of Genes
E
fold change > 1.5
### Chart
| Category | Pvalue<0.01 | Pvalue<0.05 | Pvalue<0.1 |
|---|---|---|---|
| up | 85.0 | 91.0 | 96.0 |
| down | 391.0 | 559.0 | 629.0 | P value<0.01
 P value<0.05
 P value<0.1
Number of Genes

## Slide 2
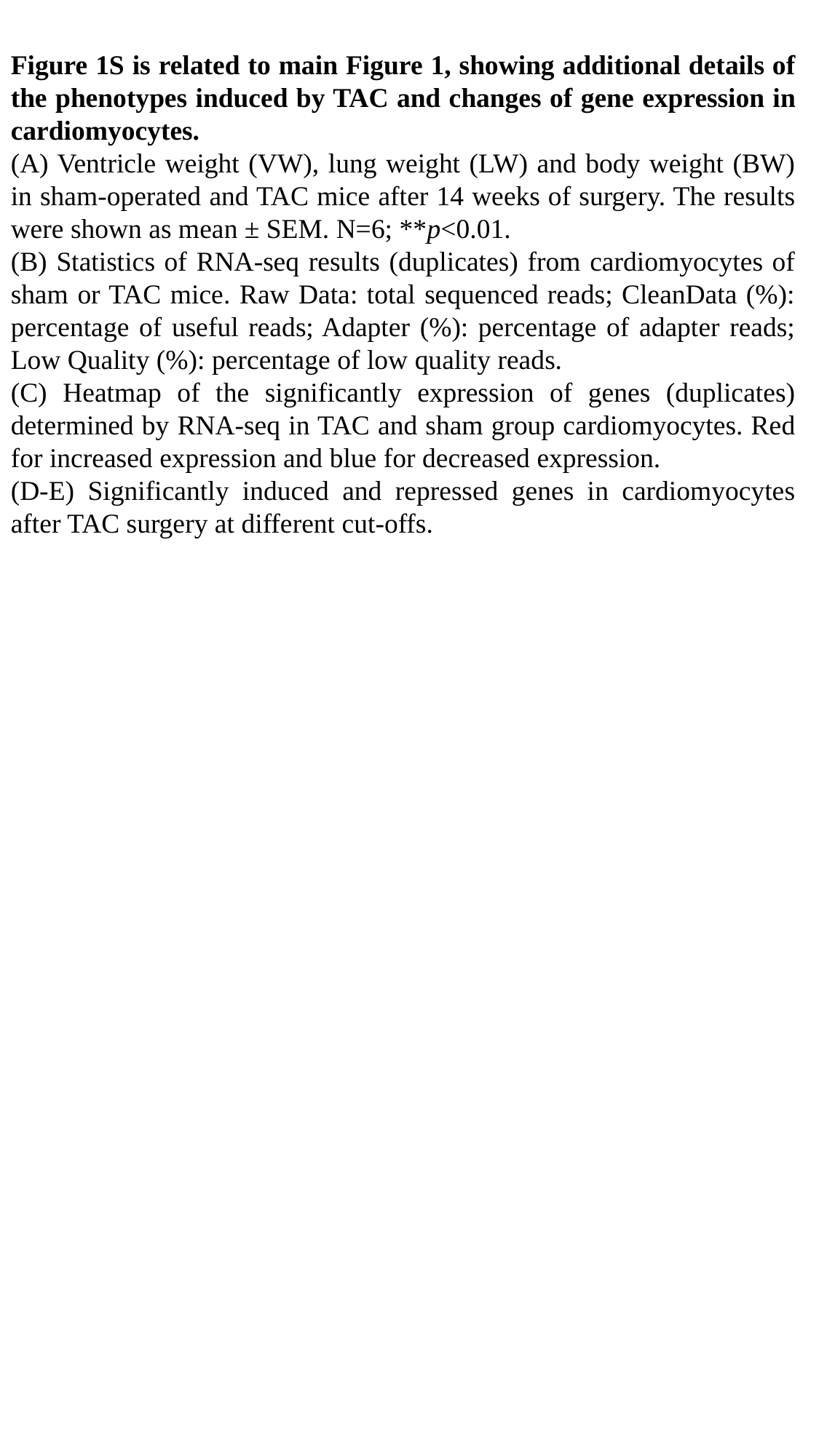

Figure 1S is related to main Figure 1, showing additional details of the phenotypes induced by TAC and changes of gene expression in cardiomyocytes.
(A) Ventricle weight (VW), lung weight (LW) and body weight (BW) in sham-operated and TAC mice after 14 weeks of surgery. The results were shown as mean ± SEM. N=6; **p<0.01.
(B) Statistics of RNA-seq results (duplicates) from cardiomyocytes of sham or TAC mice. Raw Data: total sequenced reads; CleanData (%): percentage of useful reads; Adapter (%): percentage of adapter reads; Low Quality (%): percentage of low quality reads.
(C) Heatmap of the significantly expression of genes (duplicates) determined by RNA-seq in TAC and sham group cardiomyocytes. Red for increased expression and blue for decreased expression.
(D-E) Significantly induced and repressed genes in cardiomyocytes after TAC surgery at different cut-offs.

## Slide 3
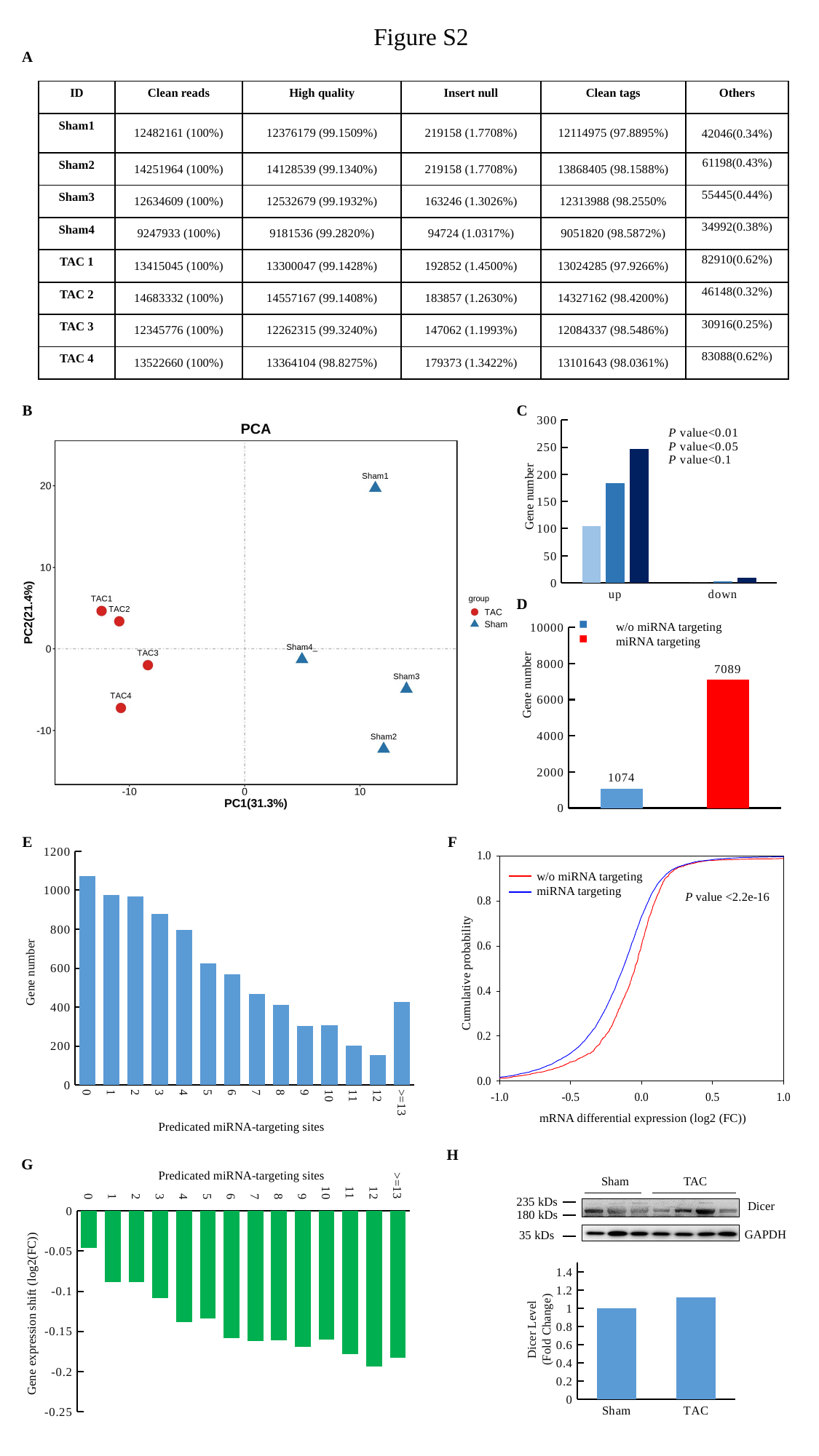

Figure S2
A
| ID | Clean reads | High quality | Insert null | Clean tags | Others |
| --- | --- | --- | --- | --- | --- |
| Sham1 | 12482161 (100%) | 12376179 (99.1509%) | 219158 (1.7708%) | 12114975 (97.8895%) | 42046(0.34%) |
| Sham2 | 14251964 (100%) | 14128539 (99.1340%) | 219158 (1.7708%) | 13868405 (98.1588%) | 61198(0.43%) |
| Sham3 | 12634609 (100%) | 12532679 (99.1932%) | 163246 (1.3026%) | 12313988 (98.2550% | 55445(0.44%) |
| Sham4 | 9247933 (100%) | 9181536 (99.2820%) | 94724 (1.0317%) | 9051820 (98.5872%) | 34992(0.38%) |
| TAC 1 | 13415045 (100%) | 13300047 (99.1428%) | 192852 (1.4500%) | 13024285 (97.9266%) | 82910(0.62%) |
| TAC 2 | 14683332 (100%) | 14557167 (99.1408%) | 183857 (1.2630%) | 14327162 (98.4200%) | 46148(0.32%) |
| TAC 3 | 12345776 (100%) | 12262315 (99.3240%) | 147062 (1.1993%) | 12084337 (98.5486%) | 30916(0.25%) |
| TAC 4 | 13522660 (100%) | 13364104 (98.8275%) | 179373 (1.3422%) | 13101643 (98.0361%) | 83088(0.62%) |
B
C
### Chart
| Category | Pvalue<0.01 | Pvalue<0.05 | Pvalue<0.1 |
|---|---|---|---|
| up | 105.0 | 184.0 | 247.0 |
| down | 0.0 | 3.0 | 9.0 |Gene number
D
 w/o miRNA targeting
 miRNA targeting
### Chart
| Category | |
|---|---|Gene number
w/o miRNA targeting
miRNA targeting
P value <2.2e-16
Cumulative probability
mRNA differential expression (log2 (FC))
E
F
### Chart
| Category | binding number |
|---|---|
| 0 | 1074.0 |
| 1 | 976.0 |
| 2 | 967.0 |
| 3 | 879.0 |
| 4 | 798.0 |
| 5 | 626.0 |
| 6 | 570.0 |
| 7 | 466.0 |
| 8 | 411.0 |
| 9 | 305.0 |
| 10 | 308.0 |
| 11 | 202.0 |
| 12 | 153.0 |
| >=13 | 428.0 |Gene number
Predicated miRNA-targeting sites
H
G
Predicated miRNA-targeting sites
### Chart
| Category | median gene changes |
|---|---|
| 0 | -0.0462184175839514 |
| 1 | -0.0883558744208468 |
| 2 | -0.0886117051580418 |
| 3 | -0.108477805814686 |
| 4 | -0.138189233428182 |
| 5 | -0.133690588643654 |
| 6 | -0.158616510903133 |
| 7 | -0.162194533965996 |
| 8 | -0.16082279433506 |
| 9 | -0.169172225510342 |
| 10 | -0.160233251916413 |
| 11 | -0.178039008354445 |
| 12 | -0.193713345329887 |
| >=13 | -0.182818820714162 |Gene expression shift (log2(FC))
Sham
TAC
235 kDs
Dicer
180 kDs
GAPDH
35 kDs
### Chart
| Category | |
|---|---|
| Sham | 1.0 |
| TAC | 1.1186560368400493 |Dicer Level
(Fold Change)

## Slide 4
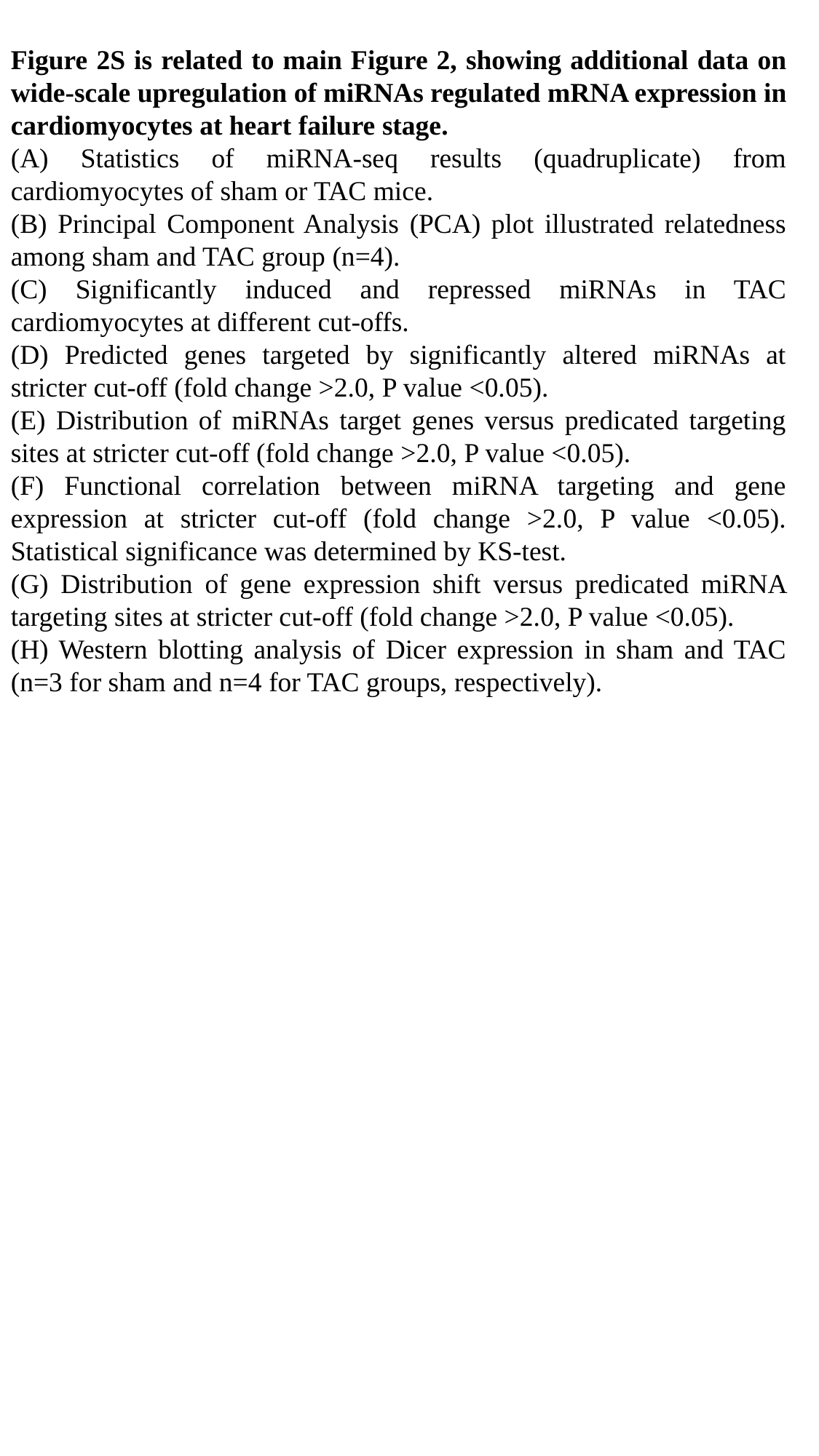

Figure 2S is related to main Figure 2, showing additional data on wide-scale upregulation of miRNAs regulated mRNA expression in cardiomyocytes at heart failure stage.
(A) Statistics of miRNA-seq results (quadruplicate) from cardiomyocytes of sham or TAC mice.
(B) Principal Component Analysis (PCA) plot illustrated relatedness among sham and TAC group (n=4).
(C) Significantly induced and repressed miRNAs in TAC cardiomyocytes at different cut-offs.
(D) Predicted genes targeted by significantly altered miRNAs at stricter cut-off (fold change >2.0, P value <0.05).
(E) Distribution of miRNAs target genes versus predicated targeting sites at stricter cut-off (fold change >2.0, P value <0.05).
(F) Functional correlation between miRNA targeting and gene expression at stricter cut-off (fold change >2.0, P value <0.05). Statistical significance was determined by KS-test.
(G) Distribution of gene expression shift versus predicated miRNA targeting sites at stricter cut-off (fold change >2.0, P value <0.05).
(H) Western blotting analysis of Dicer expression in sham and TAC (n=3 for sham and n=4 for TAC groups, respectively).

## Slide 5
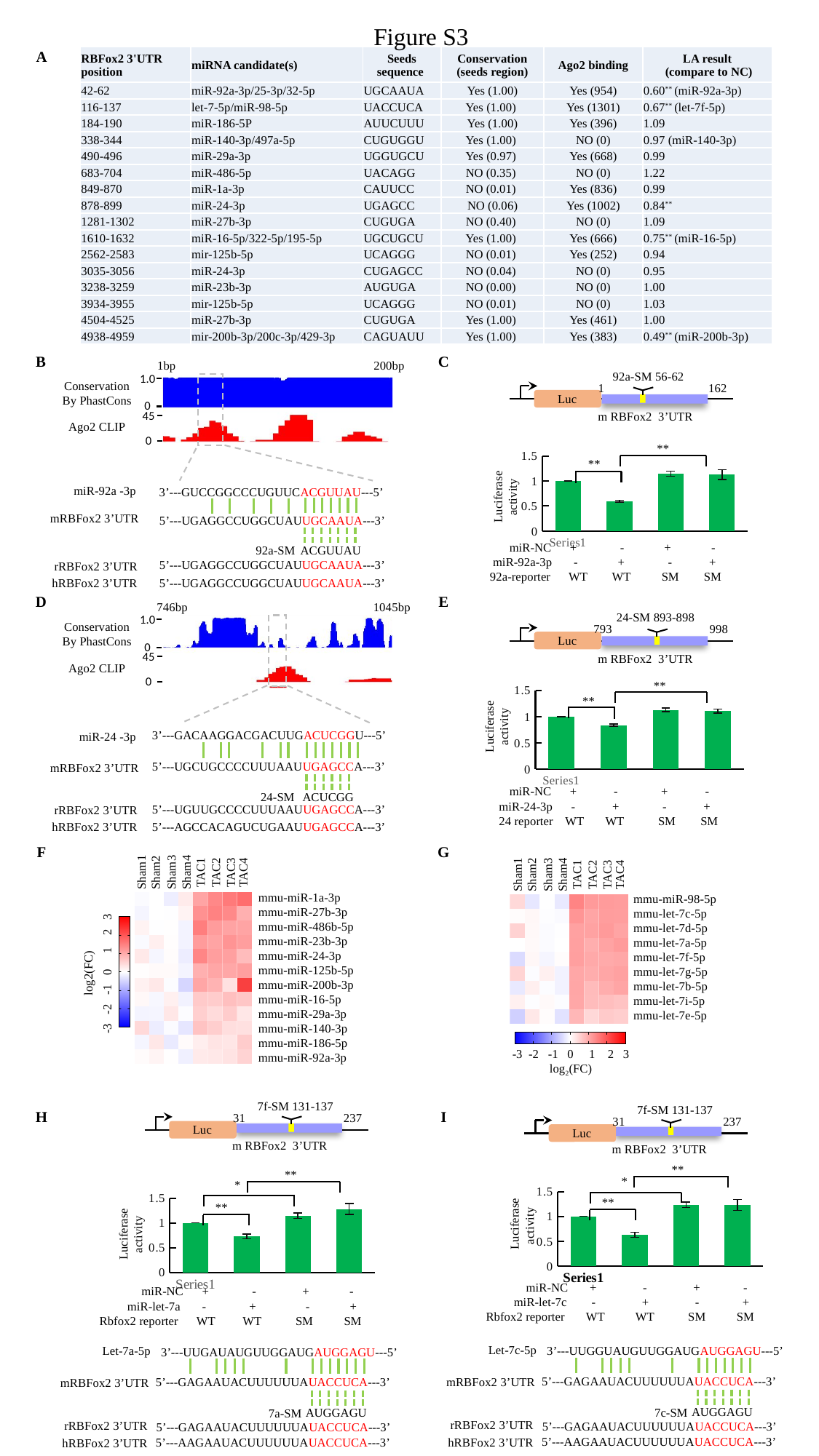

Figure S3
A
| RBFox2 3'UTR position | miRNA candidate(s) | Seeds sequence | Conservation (seeds region) | Ago2 binding | LA result (compare to NC) |
| --- | --- | --- | --- | --- | --- |
| 42-62 | miR-92a-3p/25-3p/32-5p | UGCAAUA | Yes (1.00) | Yes (954) | 0.60\*\* (miR-92a-3p) |
| 116-137 | let-7-5p/miR-98-5p | UACCUCA | Yes (1.00) | Yes (1301) | 0.67\*\* (let-7f-5p) |
| 184-190 | miR-186-5P | AUUCUUU | Yes (1.00) | Yes (396) | 1.09 |
| 338-344 | miR-140-3p/497a-5p | CUGUGGU | Yes (1.00) | NO (0) | 0.97 (miR-140-3p) |
| 490-496 | miR-29a-3p | UGGUGCU | Yes (0.97) | Yes (668) | 0.99 |
| 683-704 | miR-486-5p | UACAGG | NO (0.35) | NO (0) | 1.22 |
| 849-870 | miR-1a-3p | CAUUCC | NO (0.01) | Yes (836) | 0.99 |
| 878-899 | miR-24-3p | UGAGCC | NO (0.06) | Yes (1002) | 0.84\*\* |
| 1281-1302 | miR-27b-3p | CUGUGA | NO (0.40) | NO (0) | 1.09 |
| 1610-1632 | miR-16-5p/322-5p/195-5p | UGCUGCU | Yes (1.00) | Yes (666) | 0.75\*\* (miR-16-5p) |
| 2562-2583 | mir-125b-5p | UCAGGG | NO (0.01) | Yes (252) | 0.94 |
| 3035-3056 | miR-24-3p | CUGAGCC | NO (0.04) | NO (0) | 0.95 |
| 3238-3259 | miR-23b-3p | AUGUGA | NO (0.00) | NO (0) | 1.00 |
| 3934-3955 | mir-125b-5p | UCAGGG | NO (0.01) | NO (0) | 1.03 |
| 4504-4525 | miR-27b-3p | CUGUGA | Yes (1.00) | Yes (461) | 1.00 |
| 4938-4959 | mir-200b-3p/200c-3p/429-3p | CAGUAUU | Yes (1.00) | Yes (383) | 0.49\*\* (miR-200b-3p) |
B
C
1bp
200bp
1.0
0
Conservation
By PhastCons
45
0
Ago2 CLIP
miR-92a -3p
3’---GUCCGGCCCUGUUCACGUUAU---5’
mRBFox2 3’UTR
5’---UGAGGCCUGGCUAUUGCAAUA---3’
ACGUUAU
92a-SM
5’---UGAGGCCUGGCUAUUGCAAUA---3’
rRBFox2 3’UTR
5’---UGAGGCCUGGCUAUUGCAAUA---3’
hRBFox2 3’UTR
 92a-SM 56-62
1
162
Luc
m RBFox2 3’UTR
**
### Chart
| Category | |
|---|---|
| | 1.0 |
| | 0.5970926249750744 |
| | 1.1476694938285712 |
| | 1.1279231264562752 |**
Luciferase
 activity
 miR-NC + - + -
 miR-92a-3p - + - +
 92a-reporter WT WT SM SM
D
E
746bp
1045bp
1.0
0
Conservation
By PhastCons
45
0
Ago2 CLIP
3’---GACAAGGACGACUUGACUCGGU---5’
miR-24 -3p
5’---UGCUGCCCCUUUAAUUGAGCCA---3’
mRBFox2 3’UTR
24-SM
ACUCGG
5’---UGUUGCCCCUUUAAUUGAGCCA---3’
rRBFox2 3’UTR
hRBFox2 3’UTR
5’---AGCCACAGUCUGAAUUGAGCCA---3’
 24-SM 893-898
793
998
Luc
m RBFox2 3’UTR
**
### Chart
| Category | |
|---|---|
| | 1.0 |
| | 0.8373841463788441 |
| | 1.1255957375711183 |
| | 1.1023282193668007 |**
Luciferase
 activity
 miR-NC + - + -
 miR-24-3p - + - +
 24 reporter WT WT SM SM
F
G
Sham1
Sham2
Sham3
Sham4
TAC1
TAC2
TAC3
TAC4
mmu-miR-1a-3p
mmu-miR-27b-3p
mmu-miR-486b-5p
mmu-miR-23b-3p
mmu-miR-24-3p
mmu-miR-125b-5p
mmu-miR-200b-3p
mmu-miR-16-5p
mmu-miR-29a-3p
mmu-miR-140-3p
mmu-miR-186-5p
mmu-miR-92a-3p
-3 -2 -1 0 1 2 3
 log2(FC)
Sham1
Sham2
Sham3
Sham4
TAC1
TAC2
TAC3
TAC4
mmu-miR-98-5p
mmu-let-7c-5p
mmu-let-7d-5p
mmu-let-7a-5p
mmu-let-7f-5p
mmu-let-7g-5p
mmu-let-7b-5p
mmu-let-7i-5p
mmu-let-7e-5p
-3 -2 -1 0 1 2 3
 log2(FC)
 7f-SM 131-137
31
237
Luc
m RBFox2 3’UTR
**
*
### Chart
| Category | |
|---|---|
| | 1.0 |
| | 0.728480362057649 |
| | 1.1455622483176229 |
| | 1.2824360340949517 |**
Luciferase
 activity
 miR-NC + - + -
 miR-let-7a - + - +
Rbfox2 reporter WT WT SM SM
Let-7a-5p
3’---UUGAUAUGUUGGAUGAUGGAGU---5’
5’---GAGAAUACUUUUUUAUACCUCA---3’
mRBFox2 3’UTR
AUGGAGU
7a-SM
rRBFox2 3’UTR
5’---GAGAAUACUUUUUUAUACCUCA---3’
5’---AAGAAUACUUUUUUAUACCUCA---3’
hRBFox2 3’UTR
 7f-SM 131-137
31
237
Luc
m RBFox2 3’UTR
**
*
### Chart
| Category | |
|---|---|
| | 1.0 |
| | 0.628467 |
| | 1.2322466666666665 |
| | 1.2314776666666667 |**
 Luciferase
 activity
 miR-NC + - + -
 miR-let-7c - + - +
Rbfox2 reporter WT WT SM SM
Let-7c-5p
3’---UUGGUAUGUUGGAUGAUGGAGU---5’
5’---GAGAAUACUUUUUUAUACCUCA---3’
mRBFox2 3’UTR
AUGGAGU
7c-SM
rRBFox2 3’UTR
5’---GAGAAUACUUUUUUAUACCUCA---3’
5’---AAGAAUACUUUUUUAUACCUCA---3’
hRBFox2 3’UTR
H
I

## Slide 6
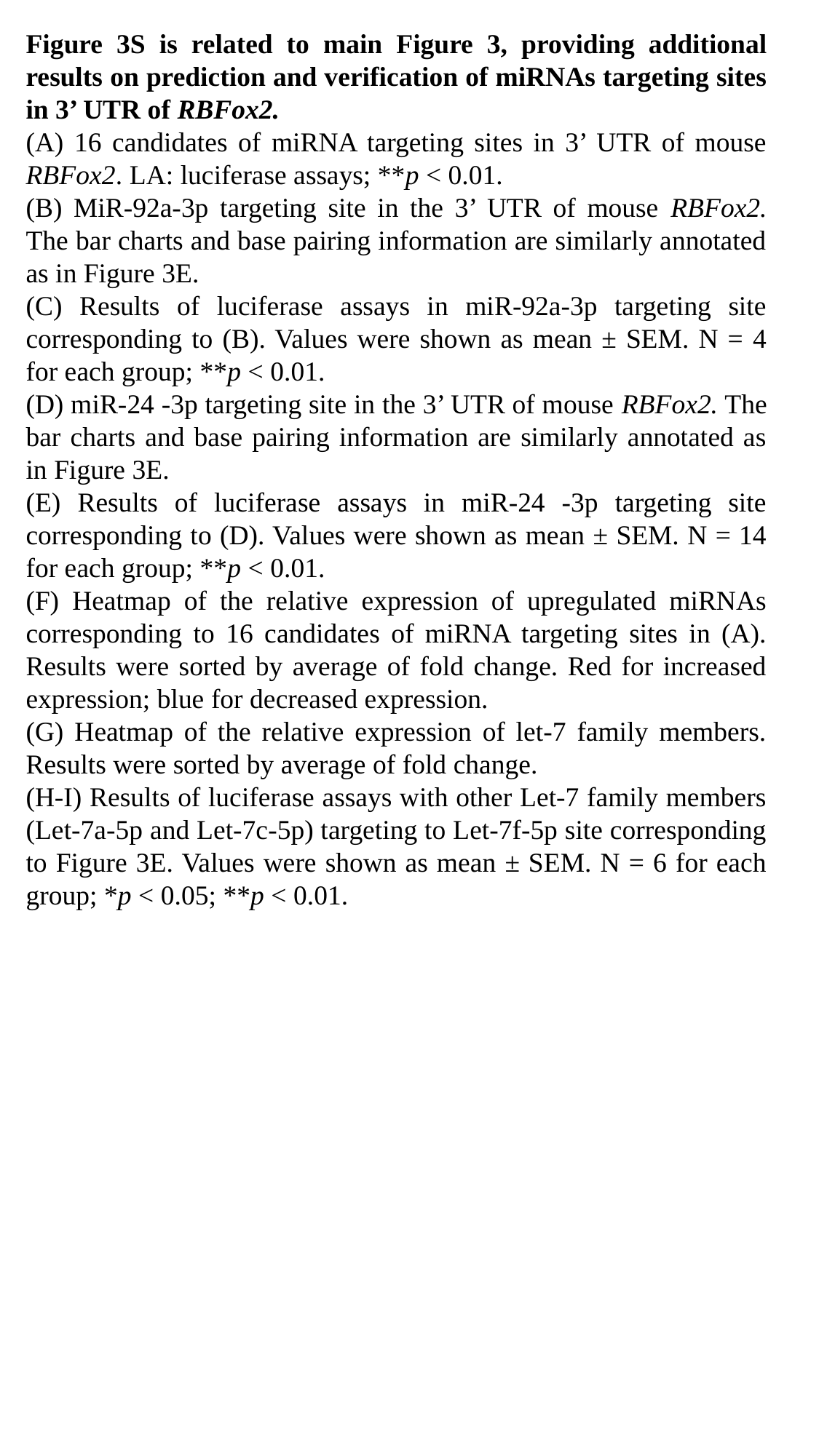

Figure 3S is related to main Figure 3, providing additional results on prediction and verification of miRNAs targeting sites in 3’ UTR of RBFox2.
(A) 16 candidates of miRNA targeting sites in 3’ UTR of mouse RBFox2. LA: luciferase assays; **p < 0.01.
(B) MiR-92a-3p targeting site in the 3’ UTR of mouse RBFox2. The bar charts and base pairing information are similarly annotated as in Figure 3E.
(C) Results of luciferase assays in miR-92a-3p targeting site corresponding to (B). Values were shown as mean ± SEM. N = 4 for each group; **p < 0.01.
(D) miR-24 -3p targeting site in the 3’ UTR of mouse RBFox2. The bar charts and base pairing information are similarly annotated as in Figure 3E.
(E) Results of luciferase assays in miR-24 -3p targeting site corresponding to (D). Values were shown as mean ± SEM. N = 14 for each group; **p < 0.01.
(F) Heatmap of the relative expression of upregulated miRNAs corresponding to 16 candidates of miRNA targeting sites in (A). Results were sorted by average of fold change. Red for increased expression; blue for decreased expression.
(G) Heatmap of the relative expression of let-7 family members. Results were sorted by average of fold change.
(H-I) Results of luciferase assays with other Let-7 family members (Let-7a-5p and Let-7c-5p) targeting to Let-7f-5p site corresponding to Figure 3E. Values were shown as mean ± SEM. N = 6 for each group; *p < 0.05; **p < 0.01.

## Slide 7
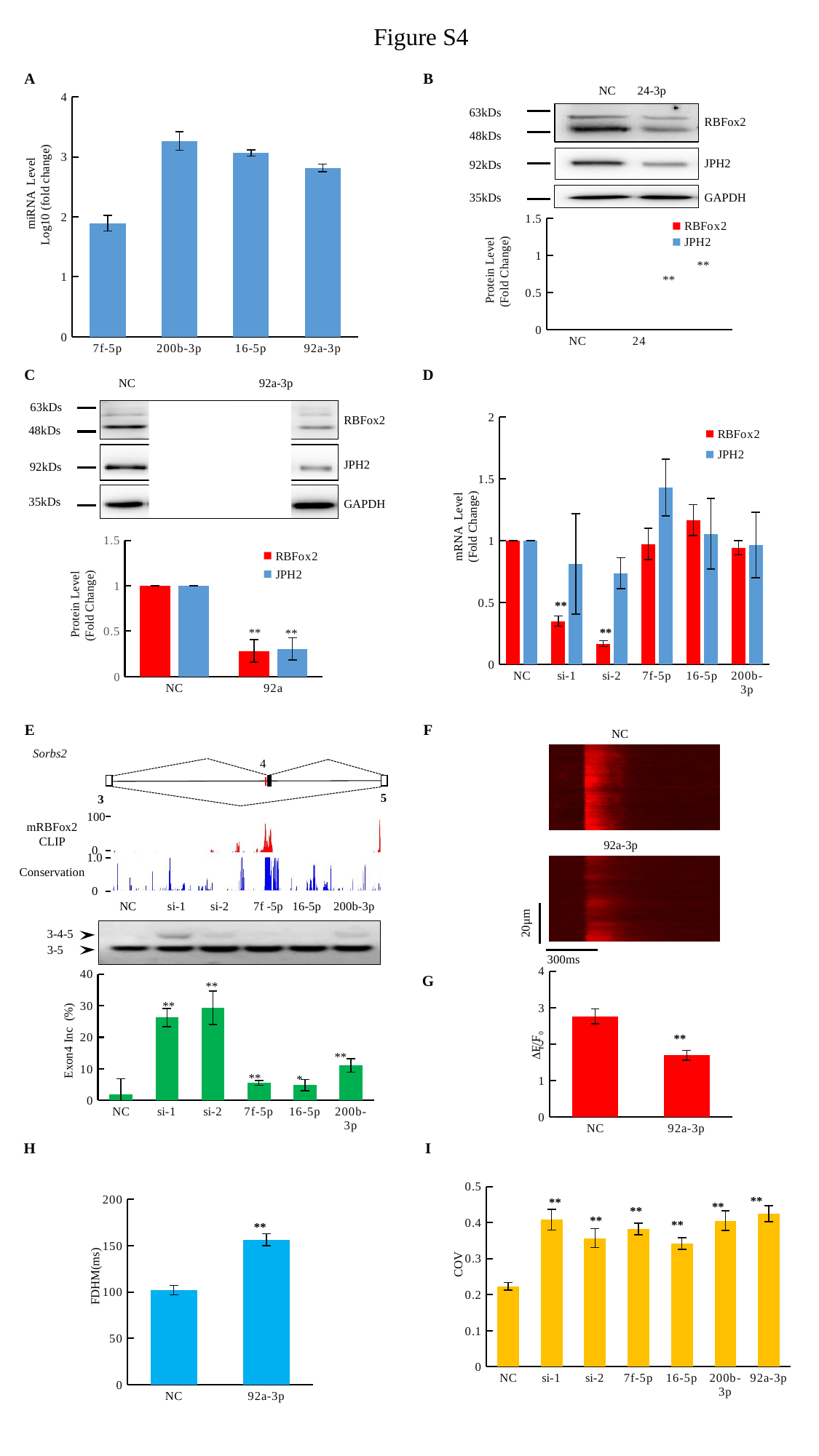

Figure S4
A
B
### Chart
| Category | |
|---|---|
| 7f-5p | 1.8926334219434067 |
| 200b-3p | 3.2657818836142076 |
| 16-5p | 3.064779653959557 |
| 92a-3p | 2.8186581028277264 | miRNA Level
Log10 (fold change)
NC 24-3p
63kDs
RBFox2
48kDs
JPH2
92kDs
35kDs
GAPDH
### Chart
| Category | | |
|---|---|---|
| NC | 1.0 | 1.0 |
| 24 | 0.4990636005179716 | 0.6901234054190392 | Protein Level
(Fold Change)
**
**
C
D
NC 92a-3p
63kDs
RBFox2
48kDs
JPH2
92kDs
35kDs
GAPDH
### Chart
| Category | | |
|---|---|---|
| NC | 1.0 | 1.0 |
| si-1 | 0.34980691536138925 | 0.8127780573071631 |
| si-2 | 0.16724981508236567 | 0.7360619488730256 |
| 7f-5p | 0.973022223066202 | 1.4288502290489582 |
| 16-5p | 1.166593160440917 | 1.054739853099224 |
| 200b-3p | 0.9426664295445604 | 0.9648337028708706 |mRNA Level
(Fold Change)
**
**
### Chart
| Category | | |
|---|---|---|
| NC | 1.0 | 1.0 |
| 92a | 0.2823026525492777 | 0.30316031980412417 | Protein Level
(Fold Change)
**
**
E
F
NC
92a-3p
20μm
300ms
Sorbs2
4
5
3
100
0
1.0
0
mRBFox2 CLIP
Conservation
NC si-1 si-2 7f -5p 16-5p 200b-3p
3-4-5
3-5
### Chart
| Category | |
|---|---|
| NC | 1.89 |
| si-1 | 26.237000000000002 |
| si-2 | 29.309000000000005 |
| 7f-5p | 5.535 |
| 16-5p | 4.858 |
| 200b-3p | 11.07 |**
**
Exon4 Inc (%)
**
**
*
### Chart
| Category | |
|---|---|
| NC | 2.7652500000000004 |
| 92a-3p | 1.6957201920686917 |G
**
ΔF/F0
H
I
### Chart
| Category | |
|---|---|
| NC | 0.22315014035737432 |
| si-1 | 0.4075474565023568 |
| si-2 | 0.3565727144599763 |
| 7f-5p | 0.3823489958932588 |
| 16-5p | 0.34172200557148485 |
| 200b-3p | 0.40491697127461584 |
| 92a-3p | 0.42414393541299095 |**
**
**
**
**
**
COV
### Chart
| Category | |
|---|---|
| NC | 102.1020515625 |
| 92a-3p | 156.50199558423913 |**
FDHM(ms)

## Slide 8
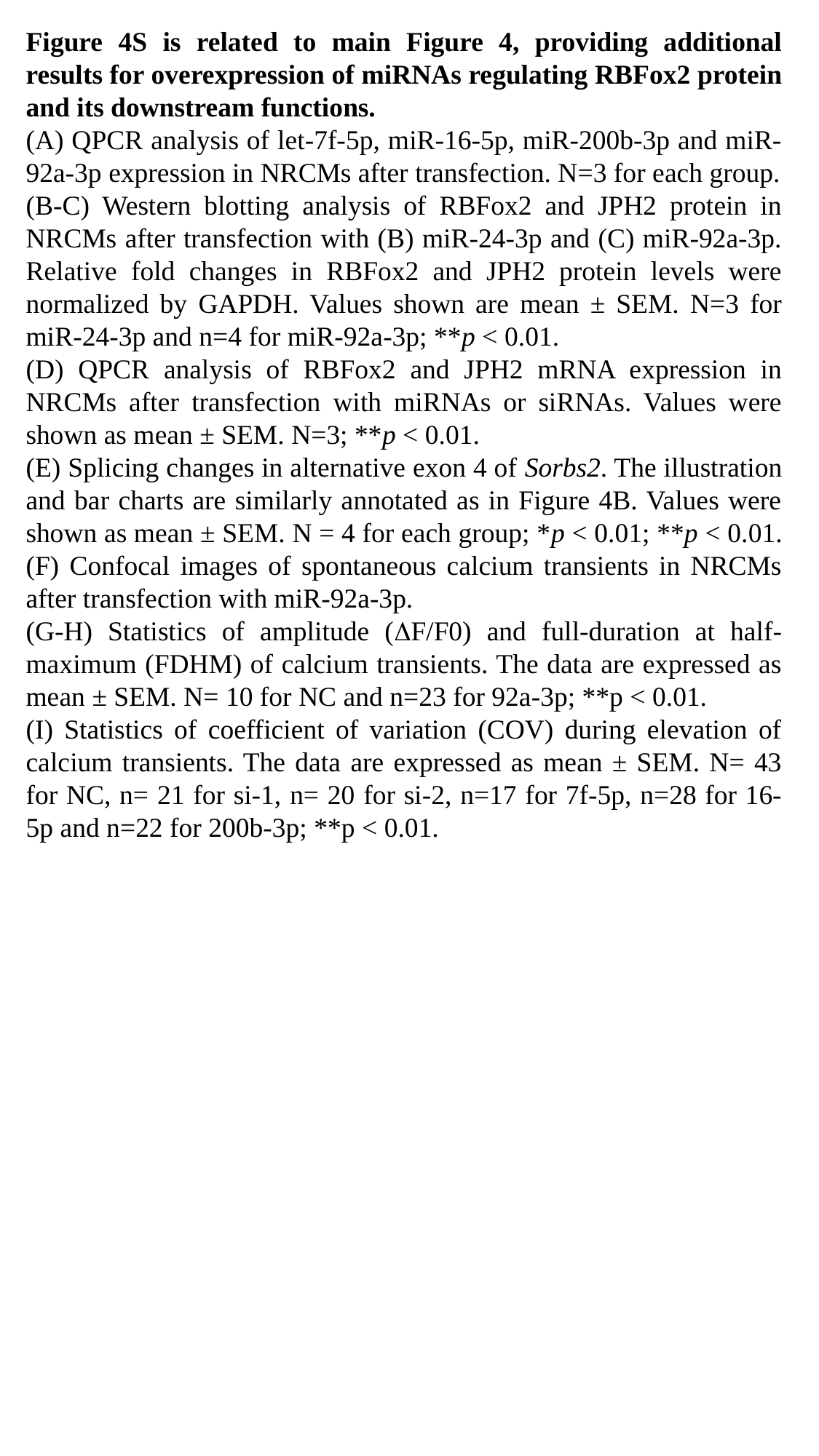

Figure 4S is related to main Figure 4, providing additional results for overexpression of miRNAs regulating RBFox2 protein and its downstream functions.
(A) QPCR analysis of let-7f-5p, miR-16-5p, miR-200b-3p and miR-92a-3p expression in NRCMs after transfection. N=3 for each group.
(B-C) Western blotting analysis of RBFox2 and JPH2 protein in NRCMs after transfection with (B) miR-24-3p and (C) miR-92a-3p. Relative fold changes in RBFox2 and JPH2 protein levels were normalized by GAPDH. Values shown are mean ± SEM. N=3 for miR-24-3p and n=4 for miR-92a-3p; **p < 0.01.
(D) QPCR analysis of RBFox2 and JPH2 mRNA expression in NRCMs after transfection with miRNAs or siRNAs. Values were shown as mean ± SEM. N=3; **p < 0.01.
(E) Splicing changes in alternative exon 4 of Sorbs2. The illustration and bar charts are similarly annotated as in Figure 4B. Values were shown as mean ± SEM. N = 4 for each group; *p < 0.01; **p < 0.01.
(F) Confocal images of spontaneous calcium transients in NRCMs after transfection with miR-92a-3p.
(G-H) Statistics of amplitude (ΔF/F0) and full-duration at half-maximum (FDHM) of calcium transients. The data are expressed as mean ± SEM. N= 10 for NC and n=23 for 92a-3p; **p < 0.01.
(I) Statistics of coefficient of variation (COV) during elevation of calcium transients. The data are expressed as mean ± SEM. N= 43 for NC, n= 21 for si-1, n= 20 for si-2, n=17 for 7f-5p, n=28 for 16-5p and n=22 for 200b-3p; **p < 0.01.

## Slide 9
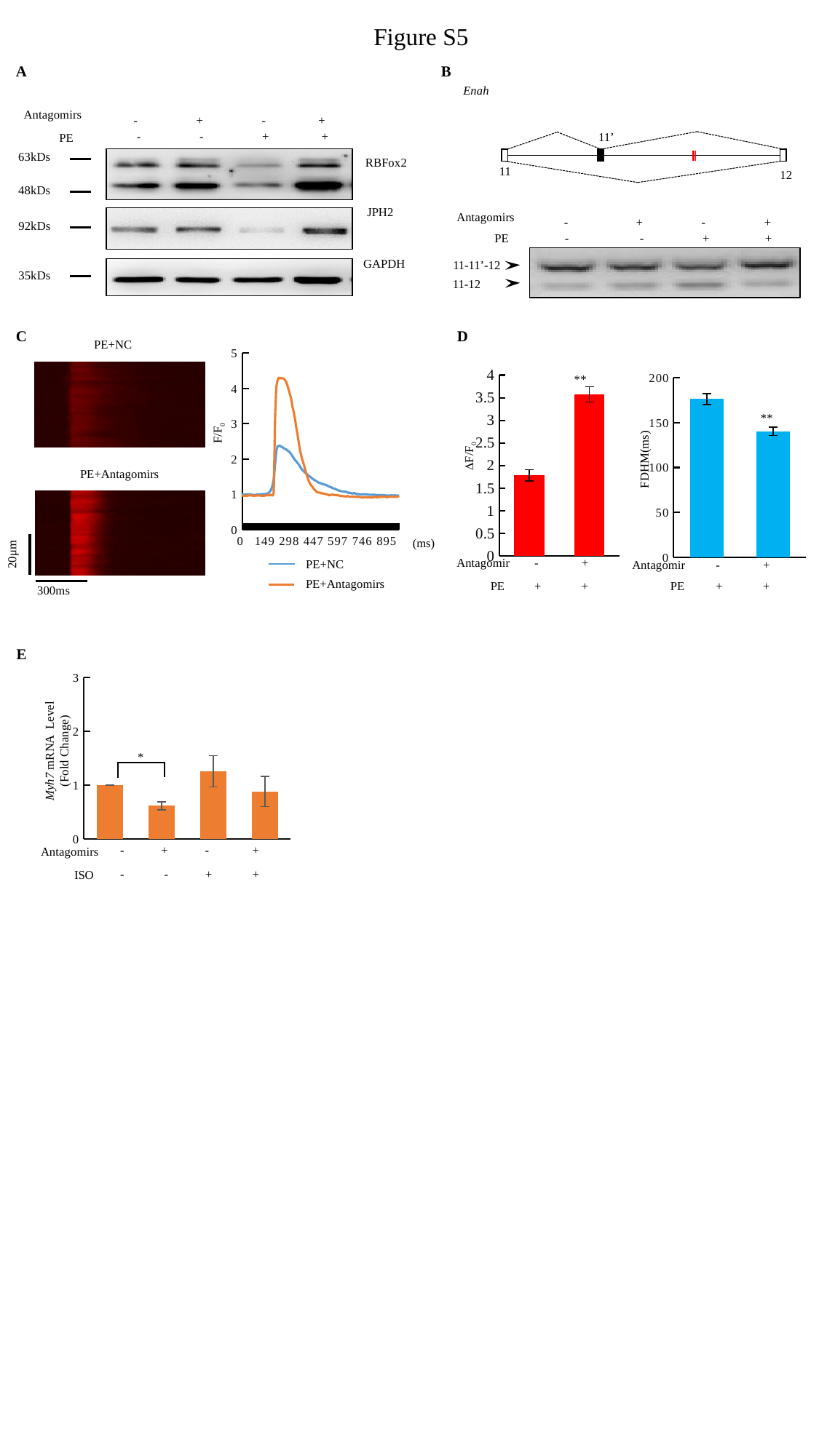

Figure S5
A
B
Enah
Antagomirs
 - + - +
- - + +
PE
63kDs
RBFox2
48kDs
JPH2
92kDs
GAPDH
35kDs
11’
11
12
Antagomirs
 - + - +
- - + +
PE
11-11’-12
11-12
C
D
PE+NC
PE+Antagomirs
20μm
300ms
### Chart
| Category | PE+NC | PE+Antigomirs |
|---|---|---|
| 0 | 1.009 | 0.965 |
| 1.9119999999999999 | 1.008 | 0.966 |
| 3.8239999999999998 | 1.005 | 0.964 |
| 5.7359999999999998 | 1.002 | 0.963 |
| 7.6479999999999997 | 0.999 | 0.962 |
| 9.56 | 0.997 | 0.961 |
| 11.472 | 0.996 | 0.961 |
| 13.384 | 0.995 | 0.961 |
| 15.295999999999999 | 0.994 | 0.961 |
| 17.207999999999998 | 0.994 | 0.961 |
| 19.12 | 0.994 | 0.96 |
| 21.032 | 0.994 | 0.959 |
| 22.943999999999999 | 0.993 | 0.958 |
| 24.856000000000002 | 0.995 | 0.958 |
| 26.768000000000001 | 0.998 | 0.959 |
| 28.68 | 1.001 | 0.961 |
| 30.591999999999999 | 1.003 | 0.962 |
| 32.503999999999998 | 1.003 | 0.963 |
| 34.415999999999997 | 0.999 | 0.965 |
| 36.328000000000003 | 0.994 | 0.966 |
| 38.24 | 0.99 | 0.968 |
| 40.152000000000001 | 0.99 | 0.97 |
| 42.064 | 0.993 | 0.974 |
| 43.975999999999999 | 0.997 | 0.977 |
| 45.887999999999998 | 1.0 | 0.981 |
| 47.8 | 1.002 | 0.981 |
| 49.712000000000003 | 1.001 | 0.977 |
| 51.624000000000002 | 0.999 | 0.974 |
| 53.536000000000001 | 0.998 | 0.97 |
| 55.448 | 0.996 | 0.969 |
| 57.36 | 0.992 | 0.969 |
| 59.271999999999998 | 0.988 | 0.97 |
| 61.183999999999997 | 0.984 | 0.97 |
| 63.095999999999997 | 0.983 | 0.969 |
| 65.007999999999996 | 0.985 | 0.965 |
| 66.92 | 0.987 | 0.962 |
| 68.831999999999994 | 0.988 | 0.958 |
| 70.744 | 0.989 | 0.958 |
| 72.656000000000006 | 0.987 | 0.96 |
| 74.567999999999998 | 0.986 | 0.963 |
| 76.48 | 0.985 | 0.965 |
| 78.391999999999996 | 0.985 | 0.967 |
| 80.304000000000002 | 0.987 | 0.968 |
| 82.215999999999994 | 0.989 | 0.968 |
| 84.128 | 0.991 | 0.969 |
| 86.04 | 0.992 | 0.97 |
| 87.951999999999998 | 0.994 | 0.969 |
| 89.864000000000004 | 0.996 | 0.969 |
| 91.775999999999996 | 0.998 | 0.969 |
| 93.688000000000002 | 0.998 | 0.969 |
| 95.6 | 0.998 | 0.97 |
| 97.512 | 0.998 | 0.971 |
| 99.424000000000007 | 0.997 | 0.971 |
| 101.336 | 0.998 | 0.971 |
| 103.248 | 0.999 | 0.971 |
| 105.16 | 1.0 | 0.97 |
| 107.072 | 1.001 | 0.969 |
| 108.98399999999999 | 1.001 | 0.968 |
| 110.896 | 1.002 | 0.967 |
| 112.80800000000001 | 1.003 | 0.966 |
| 114.72 | 1.004 | 0.965 |
| 116.63200000000001 | 1.004 | 0.964 |
| 118.544 | 1.004 | 0.965 |
| 120.456 | 1.004 | 0.965 |
| 122.36799999999999 | 1.004 | 0.965 |
| 124.28 | 1.004 | 0.964 |
| 126.19199999999999 | 1.006 | 0.962 |
| 128.10400000000001 | 1.008 | 0.959 |
| 130.01599999999999 | 1.009 | 0.956 |
| 131.928 | 1.01 | 0.957 |
| 133.84 | 1.011 | 0.961 |
| 135.75200000000001 | 1.011 | 0.965 |
| 137.66399999999999 | 1.012 | 0.969 |
| 139.57599999999999 | 1.013 | 0.971 |
| 141.488 | 1.015 | 0.971 |
| 143.4 | 1.016 | 0.971 |
| 145.31200000000001 | 1.018 | 0.971 |
| 147.22399999999999 | 1.02 | 0.972 |
| 149.136 | 1.021 | 0.975 |
| 151.048 | 1.022 | 0.977 |
| 152.96 | 1.024 | 0.98 |
| 154.87200000000001 | 1.027 | 0.981 |
| 156.78399999999999 | 1.032 | 0.98 |
| 158.696 | 1.038 | 0.979 |
| 160.608 | 1.043 | 0.978 |
| 162.52000000000001 | 1.05 | 0.978 |
| 164.43199999999999 | 1.059 | 0.98 |
| 166.34399999999999 | 1.068 | 0.981 |
| 168.256 | 1.076 | 0.982 |
| 170.16800000000001 | 1.09 | 0.983 |
| 172.08 | 1.107 | 0.986 |
| 173.99199999999999 | 1.124 | 0.988 |
| 175.904 | 1.141 | 0.99 |
| 177.816 | 1.162 | 0.987 |
| 179.72800000000001 | 1.187 | 0.98 |
| 181.64 | 1.212 | 0.973 |
| 183.55199999999999 | 1.237 | 0.965 |
| 185.464 | 1.274 | 0.972 |
| 187.376 | 1.322 | 0.994 |
| 189.28800000000001 | 1.37 | 1.016 |
| 191.2 | 1.419 | 1.038 |
| 193.11199999999999 | 1.491 | 1.313 |
| 195.024 | 1.588 | 1.84 |
| 196.93600000000001 | 1.684 | 2.368 |
| 198.84800000000001 | 1.78 | 2.895 |
| 200.76 | 1.878 | 3.269 |
| 202.672 | 1.978 | 3.49 |
| 204.584 | 2.077 | 3.711 |
| 206.49600000000001 | 2.176 | 3.932 |
| 208.40799999999999 | 2.243 | 4.067 |
| 210.32 | 2.277 | 4.115 |
| 212.232 | 2.311 | 4.164 |
| 214.14400000000001 | 2.346 | 4.212 |
| 216.05600000000001 | 2.364 | 4.245 |
| 217.96799999999999 | 2.367 | 4.263 |
| 219.88 | 2.37 | 4.281 |
| 221.792 | 2.373 | 4.299 |
| 223.70400000000001 | 2.374 | 4.304 |
| 225.61600000000001 | 2.375 | 4.297 |
| 227.52799999999999 | 2.375 | 4.29 |
| 229.44 | 2.375 | 4.283 |
| 231.352 | 2.372 | 4.282 |
| 233.26400000000001 | 2.365 | 4.287 |
| 235.17599999999999 | 2.358 | 4.291 |
| 237.08799999999999 | 2.351 | 4.295 |
| 239 | 2.346 | 4.296 |
| 240.91200000000001 | 2.344 | 4.292 |
| 242.82400000000001 | 2.341 | 4.288 |
| 244.73599999999999 | 2.338 | 4.284 |
| 246.648 | 2.333 | 4.282 |
| 248.56 | 2.325 | 4.282 |
| 250.47200000000001 | 2.317 | 4.282 |
| 252.38399999999999 | 2.309 | 4.282 |
| 254.29599999999999 | 2.304 | 4.275 |
| 256.20800000000003 | 2.3 | 4.262 |
| 258.12 | 2.297 | 4.248 |
| 260.03199999999998 | 2.293 | 4.234 |
| 261.94400000000002 | 2.289 | 4.222 |
| 263.85599999999999 | 2.284 | 4.211 |
| 265.76799999999997 | 2.279 | 4.199 |
| 267.68 | 2.274 | 4.188 |
| 269.59199999999998 | 2.267 | 4.169 |
| 271.50400000000002 | 2.26 | 4.141 |
| 273.416 | 2.252 | 4.113 |
| 275.32799999999997 | 2.244 | 4.086 |
| 277.24 | 2.238 | 4.057 |
| 279.15199999999999 | 2.233 | 4.027 |
| 281.06400000000002 | 2.229 | 3.997 |
| 282.976 | 2.224 | 3.967 |
| 284.88799999999998 | 2.216 | 3.936 |
| 286.8 | 2.206 | 3.904 |
| 288.71199999999999 | 2.195 | 3.872 |
| 290.62400000000002 | 2.185 | 3.84 |
| 292.536 | 2.174 | 3.806 |
| 294.44799999999998 | 2.163 | 3.771 |
| 296.36 | 2.152 | 3.735 |
| 298.27199999999999 | 2.141 | 3.7 |
| 300.18400000000003 | 2.129 | 3.656 |
| 302.096 | 2.115 | 3.604 |
| 304.00799999999998 | 2.1 | 3.551 |
| 305.92 | 2.086 | 3.499 |
| 307.83199999999999 | 2.073 | 3.453 |
| 309.74400000000003 | 2.06 | 3.413 |
| 311.65600000000001 | 2.047 | 3.374 |
| 313.56799999999998 | 2.034 | 3.335 |
| 315.48 | 2.02 | 3.292 |
| 317.392 | 2.005 | 3.246 |
| 319.30399999999997 | 1.99 | 3.2 |
| 321.21600000000001 | 1.975 | 3.154 |
| 323.12799999999999 | 1.964 | 3.1 |
| 325.04000000000002 | 1.957 | 3.038 |
| 326.952 | 1.95 | 2.976 |
| 328.86399999999998 | 1.943 | 2.914 |
| 330.77600000000001 | 1.933 | 2.859 |
| 332.68799999999999 | 1.92 | 2.811 |
| 334.6 | 1.907 | 2.763 |
| 336.512 | 1.895 | 2.716 |
| 338.42399999999998 | 1.884 | 2.663 |
| 340.33600000000001 | 1.874 | 2.605 |
| 342.24799999999999 | 1.865 | 2.547 |
| 344.16 | 1.856 | 2.489 |
| 346.072 | 1.843 | 2.434 |
| 347.98399999999998 | 1.827 | 2.379 |
| 349.89600000000002 | 1.811 | 2.325 |
| 351.80799999999999 | 1.795 | 2.271 |
| 353.72 | 1.78 | 2.225 |
| 355.63200000000001 | 1.766 | 2.187 |
| 357.54399999999998 | 1.753 | 2.149 |
| 359.45600000000002 | 1.739 | 2.112 |
| 361.36799999999999 | 1.727 | 2.076 |
| 363.28 | 1.715 | 2.042 |
| 365.19200000000001 | 1.704 | 2.008 |
| 367.10399999999998 | 1.692 | 1.974 |
| 369.01600000000002 | 1.682 | 1.944 |
| 370.928 | 1.674 | 1.916 |
| 372.84 | 1.665 | 1.889 |
| 374.75200000000001 | 1.657 | 1.861 |
| 376.66399999999999 | 1.648 | 1.832 |
| 378.57600000000002 | 1.639 | 1.8 |
| 380.488 | 1.63 | 1.769 |
| 382.4 | 1.62 | 1.737 |
| 384.31200000000001 | 1.612 | 1.705 |
| 386.22399999999999 | 1.606 | 1.672 |
| 388.13600000000002 | 1.599 | 1.639 |
| 390.048 | 1.593 | 1.606 |
| 391.96 | 1.585 | 1.575 |
| 393.87200000000001 | 1.577 | 1.546 |
| 395.78399999999999 | 1.569 | 1.518 |
| 397.69600000000003 | 1.56 | 1.489 |
| 399.608 | 1.552 | 1.463 |
| 401.52 | 1.544 | 1.439 |
| 403.43200000000002 | 1.537 | 1.415 |
| 405.34399999999999 | 1.529 | 1.391 |
| 407.25599999999997 | 1.522 | 1.37 |
| 409.16800000000001 | 1.517 | 1.35 |
| 411.08 | 1.511 | 1.33 |
| 412.99200000000002 | 1.506 | 1.31 |
| 414.904 | 1.499 | 1.294 |
| 416.81599999999997 | 1.491 | 1.281 |
| 418.72800000000001 | 1.483 | 1.268 |
| 420.64 | 1.475 | 1.255 |
| 422.55200000000002 | 1.467 | 1.244 |
| 424.464 | 1.46 | 1.233 |
| 426.37599999999998 | 1.453 | 1.223 |
| 428.28800000000001 | 1.446 | 1.212 |
| 430.2 | 1.439 | 1.201 |
| 432.11200000000002 | 1.433 | 1.188 |
| 434.024 | 1.427 | 1.175 |
| 435.93599999999998 | 1.421 | 1.163 |
| 437.84800000000001 | 1.415 | 1.152 |
| 439.76 | 1.409 | 1.142 |
| 441.67200000000003 | 1.404 | 1.133 |
| 443.584 | 1.398 | 1.123 |
| 445.49599999999998 | 1.393 | 1.113 |
| 447.40800000000002 | 1.39 | 1.103 |
| 449.32 | 1.386 | 1.093 |
| 451.23200000000003 | 1.383 | 1.083 |
| 453.14400000000001 | 1.377 | 1.076 |
| 455.05599999999998 | 1.369 | 1.07 |
| 456.96800000000002 | 1.361 | 1.065 |
| 458.88 | 1.353 | 1.06 |
| 460.79199999999997 | 1.347 | 1.056 |
| 462.70400000000001 | 1.343 | 1.055 |
| 464.61599999999999 | 1.339 | 1.054 |
| 466.52800000000002 | 1.336 | 1.053 |
| 468.44 | 1.332 | 1.053 |
| 470.35199999999998 | 1.33 | 1.052 |
| 472.26400000000001 | 1.327 | 1.052 |
| 474.17599999999999 | 1.325 | 1.052 |
| 476.08800000000002 | 1.321 | 1.049 |
| 478 | 1.316 | 1.045 |
| 479.91199999999998 | 1.311 | 1.04 |
| 481.82400000000001 | 1.307 | 1.036 |
| 483.73599999999999 | 1.303 | 1.033 |
| 485.64800000000002 | 1.301 | 1.031 |
| 487.56 | 1.298 | 1.03 |
| 489.47199999999998 | 1.296 | 1.028 |
| 491.38400000000001 | 1.293 | 1.027 |
| 493.29599999999999 | 1.29 | 1.025 |
| 495.20800000000003 | 1.287 | 1.024 |
| 497.12 | 1.284 | 1.022 |
| 499.03199999999998 | 1.282 | 1.02 |
| 500.94400000000002 | 1.28 | 1.019 |
| 502.85599999999999 | 1.279 | 1.017 |
| 504.76799999999997 | 1.278 | 1.016 |
| 506.68 | 1.276 | 1.014 |
| 508.59199999999998 | 1.274 | 1.011 |
| 510.50400000000002 | 1.272 | 1.009 |
| 512.41600000000005 | 1.27 | 1.007 |
| 514.32799999999997 | 1.266 | 1.004 |
| 516.24 | 1.26 | 1.002 |
| 518.15200000000004 | 1.253 | 1.001 |
| 520.06399999999996 | 1.246 | 0.999 |
| 521.976 | 1.242 | 0.995 |
| 523.88800000000003 | 1.239 | 0.991 |
| 525.79999999999995 | 1.236 | 0.987 |
| 527.71199999999999 | 1.233 | 0.982 |
| 529.62400000000002 | 1.229 | 0.979 |
| 531.53599999999994 | 1.223 | 0.978 |
| 533.44799999999998 | 1.218 | 0.977 |
| 535.36 | 1.212 | 0.976 |
| 537.27200000000005 | 1.209 | 0.977 |
| 539.18399999999997 | 1.208 | 0.981 |
| 541.096 | 1.207 | 0.984 |
| 543.00800000000004 | 1.206 | 0.988 |
| 544.91999999999996 | 1.202 | 0.991 |
| 546.83199999999999 | 1.194 | 0.992 |
| 548.74400000000003 | 1.187 | 0.994 |
| 550.65599999999995 | 1.18 | 0.996 |
| 552.56799999999998 | 1.176 | 0.995 |
| 554.48 | 1.174 | 0.992 |
| 556.39200000000005 | 1.173 | 0.989 |
| 558.30399999999997 | 1.171 | 0.987 |
| 560.21600000000001 | 1.168 | 0.985 |
| 562.12800000000004 | 1.165 | 0.985 |
| 564.04 | 1.161 | 0.985 |
| 565.952 | 1.158 | 0.984 |
| 567.86400000000003 | 1.154 | 0.983 |
| 569.77599999999995 | 1.151 | 0.982 |
| 571.68799999999999 | 1.147 | 0.981 |
| 573.6 | 1.143 | 0.98 |
| 575.51199999999994 | 1.139 | 0.98 |
| 577.42399999999998 | 1.134 | 0.979 |
| 579.33600000000001 | 1.129 | 0.979 |
| 581.24800000000005 | 1.124 | 0.979 |
| 583.16 | 1.121 | 0.978 |
| 585.072 | 1.118 | 0.976 |
| 586.98400000000004 | 1.115 | 0.974 |
| 588.89599999999996 | 1.112 | 0.972 |
| 590.80799999999999 | 1.11 | 0.97 |
| 592.72 | 1.107 | 0.967 |
| 594.63199999999995 | 1.104 | 0.963 |
| 596.54399999999998 | 1.101 | 0.96 |
| 598.45600000000002 | 1.098 | 0.958 |
| 600.36800000000005 | 1.096 | 0.957 |
| 602.28 | 1.094 | 0.957 |
| 604.19200000000001 | 1.092 | 0.956 |
| 606.10400000000004 | 1.09 | 0.955 |
| 608.01599999999996 | 1.088 | 0.955 |
| 609.928 | 1.085 | 0.954 |
| 611.84 | 1.083 | 0.954 |
| 613.75199999999995 | 1.082 | 0.953 |
| 615.66399999999999 | 1.082 | 0.951 |
| 617.57600000000002 | 1.082 | 0.95 |
| 619.48800000000006 | 1.082 | 0.948 |
| 621.4 | 1.081 | 0.947 |
| 623.31200000000001 | 1.08 | 0.946 |
| 625.22400000000005 | 1.079 | 0.946 |
| 627.13599999999997 | 1.078 | 0.945 |
| 629.048 | 1.076 | 0.944 |
| 630.96 | 1.074 | 0.944 |
| 632.87199999999996 | 1.072 | 0.944 |
| 634.78399999999999 | 1.07 | 0.944 |
| 636.69600000000003 | 1.067 | 0.945 |
| 638.60799999999995 | 1.064 | 0.948 |
| 640.52 | 1.062 | 0.951 |
| 642.43200000000002 | 1.059 | 0.954 |
| 644.34400000000005 | 1.055 | 0.953 |
| 646.25599999999997 | 1.05 | 0.949 |
| 648.16800000000001 | 1.045 | 0.945 |
| 650.08000000000004 | 1.04 | 0.941 |
| 651.99199999999996 | 1.039 | 0.939 |
| 653.904 | 1.043 | 0.937 |
| 655.81600000000003 | 1.046 | 0.936 |
| 657.72799999999995 | 1.049 | 0.934 |
| 659.64 | 1.049 | 0.935 |
| 661.55200000000002 | 1.044 | 0.937 |
| 663.46400000000006 | 1.04 | 0.939 |
| 665.37599999999998 | 1.036 | 0.942 |
| 667.28800000000001 | 1.033 | 0.942 |
| 669.2 | 1.03 | 0.941 |
| 671.11199999999997 | 1.028 | 0.939 |
| 673.024 | 1.025 | 0.938 |
| 674.93600000000004 | 1.026 | 0.936 |
| 676.84799999999996 | 1.03 | 0.935 |
| 678.76 | 1.034 | 0.933 |
| 680.67200000000003 | 1.038 | 0.932 |
| 682.58399999999995 | 1.038 | 0.932 |
| 684.49599999999998 | 1.035 | 0.936 |
| 686.40800000000002 | 1.031 | 0.939 |
| 688.32 | 1.027 | 0.942 |
| 690.23199999999997 | 1.025 | 0.942 |
| 692.14400000000001 | 1.024 | 0.937 |
| 694.05600000000004 | 1.023 | 0.932 |
| 695.96799999999996 | 1.022 | 0.927 |
| 697.88 | 1.02 | 0.926 |
| 699.79200000000003 | 1.016 | 0.929 |
| 701.70399999999995 | 1.012 | 0.932 |
| 703.61599999999999 | 1.007 | 0.935 |
| 705.52800000000002 | 1.006 | 0.935 |
| 707.44 | 1.007 | 0.933 |
| 709.35199999999998 | 1.008 | 0.93 |
| 711.26400000000001 | 1.009 | 0.927 |
| 713.17600000000004 | 1.008 | 0.926 |
| 715.08799999999997 | 1.006 | 0.926 |
| 717 | 1.003 | 0.925 |
| 718.91200000000003 | 1.001 | 0.925 |
| 720.82399999999996 | 1.0 | 0.923 |
| 722.73599999999999 | 1.0 | 0.92 |
| 724.64800000000002 | 1.0 | 0.917 |
| 726.56 | 1.0 | 0.914 |
| 728.47199999999998 | 1.0 | 0.912 |
| 730.38400000000001 | 1.002 | 0.912 |
| 732.29600000000005 | 1.003 | 0.912 |
| 734.20799999999997 | 1.004 | 0.911 |
| 736.12 | 1.004 | 0.912 |
| 738.03200000000004 | 1.004 | 0.913 |
| 739.94399999999996 | 1.003 | 0.914 |
| 741.85599999999999 | 1.002 | 0.916 |
| 743.76800000000003 | 1.001 | 0.917 |
| 745.68 | 1.0 | 0.918 |
| 747.59199999999998 | 1.0 | 0.919 |
| 749.50400000000002 | 0.999 | 0.919 |
| 751.41600000000005 | 1.0 | 0.92 |
| 753.32799999999997 | 1.001 | 0.92 |
| 755.24 | 1.003 | 0.92 |
| 757.15200000000004 | 1.005 | 0.92 |
| 759.06399999999996 | 1.005 | 0.919 |
| 760.976 | 1.002 | 0.918 |
| 762.88800000000003 | 0.999 | 0.917 |
| 764.8 | 0.996 | 0.916 |
| 766.71199999999999 | 0.994 | 0.916 |
| 768.62400000000002 | 0.994 | 0.918 |
| 770.53599999999994 | 0.994 | 0.92 |
| 772.44799999999998 | 0.993 | 0.921 |
| 774.36 | 0.993 | 0.921 |
| 776.27200000000005 | 0.993 | 0.918 |
| 778.18399999999997 | 0.993 | 0.914 |
| 780.096 | 0.993 | 0.911 |
| 782.00800000000004 | 0.992 | 0.911 |
| 783.92 | 0.99 | 0.914 |
| 785.83199999999999 | 0.989 | 0.917 |
| 787.74400000000003 | 0.987 | 0.92 |
| 789.65599999999995 | 0.987 | 0.92 |
| 791.56799999999998 | 0.988 | 0.916 |
| 793.48 | 0.989 | 0.912 |
| 795.39200000000005 | 0.99 | 0.908 |
| 797.30399999999997 | 0.99 | 0.909 |
| 799.21600000000001 | 0.99 | 0.915 |
| 801.12800000000004 | 0.989 | 0.92 |
| 803.04 | 0.989 | 0.926 |
| 804.952 | 0.989 | 0.929 |
| 806.86400000000003 | 0.989 | 0.929 |
| 808.77599999999995 | 0.989 | 0.93 |
| 810.68799999999999 | 0.988 | 0.93 |
| 812.6 | 0.988 | 0.928 |
| 814.51199999999994 | 0.987 | 0.924 |
| 816.42399999999998 | 0.987 | 0.92 |
| 818.33600000000001 | 0.986 | 0.917 |
| 820.24800000000005 | 0.985 | 0.915 |
| 822.16 | 0.984 | 0.915 |
| 824.072 | 0.983 | 0.915 |
| 825.98400000000004 | 0.983 | 0.916 |
| 827.89599999999996 | 0.982 | 0.918 |
| 829.80799999999999 | 0.982 | 0.923 |
| 831.72 | 0.982 | 0.927 |
| 833.63199999999995 | 0.982 | 0.931 |
| 835.54399999999998 | 0.982 | 0.932 |
| 837.45600000000002 | 0.983 | 0.93 |
| 839.36800000000005 | 0.984 | 0.927 |
| 841.28 | 0.985 | 0.924 |
| 843.19200000000001 | 0.984 | 0.925 |
| 845.10400000000004 | 0.983 | 0.931 |
| 847.01599999999996 | 0.981 | 0.936 |
| 848.928 | 0.979 | 0.942 |
| 850.84 | 0.979 | 0.942 |
| 852.75199999999995 | 0.979 | 0.939 |
| 854.66399999999999 | 0.979 | 0.935 |
| 856.57600000000002 | 0.98 | 0.931 |
| 858.48800000000006 | 0.979 | 0.929 |
| 860.4 | 0.978 | 0.931 |
| 862.31200000000001 | 0.977 | 0.932 |
| 864.22400000000005 | 0.976 | 0.933 |
| 866.13599999999997 | 0.975 | 0.934 |
| 868.048 | 0.975 | 0.933 |
| 869.96 | 0.975 | 0.933 |
| 871.87199999999996 | 0.975 | 0.932 |
| 873.78399999999999 | 0.975 | 0.931 |
| 875.69600000000003 | 0.973 | 0.928 |
| 877.60799999999995 | 0.972 | 0.926 |
| 879.52 | 0.97 | 0.924 |
| 881.43200000000002 | 0.969 | 0.922 |
| 883.34400000000005 | 0.969 | 0.923 |
| 885.25599999999997 | 0.968 | 0.923 |
| 887.16800000000001 | 0.968 | 0.923 |
| 889.08 | 0.968 | 0.925 |
| 890.99199999999996 | 0.968 | 0.927 |
| 892.904 | 0.969 | 0.929 |
| 894.81600000000003 | 0.969 | 0.932 |
| 896.72799999999995 | 0.969 | 0.933 |
| 898.64 | 0.969 | 0.933 |
| 900.55200000000002 | 0.969 | 0.934 |
| 902.46400000000006 | 0.969 | 0.934 |
| 904.37599999999998 | 0.97 | 0.935 |
| 906.28800000000001 | 0.972 | 0.934 |
| 908.2 | 0.973 | 0.934 |
| 910.11199999999997 | 0.974 | 0.934 |
| 912.024 | 0.975 | 0.935 |
| 913.93600000000004 | 0.973 | 0.936 |
| 915.84799999999996 | 0.972 | 0.937 |
| 917.76 | 0.971 | 0.938 |
| 919.67200000000003 | 0.971 | 0.937 |
| 921.58399999999995 | 0.971 | 0.935 |
| 923.49599999999998 | 0.971 | 0.933 |
| 925.40800000000002 | 0.971 | 0.93 |
| 927.32 | 0.972 | 0.93 |
| 929.23199999999997 | 0.974 | 0.93 |
| 931.14400000000001 | 0.975 | 0.931 |
| 933.05600000000004 | 0.977 | 0.931 |
| 934.96799999999996 | 0.976 | 0.931 |
| 936.88 | 0.973 | 0.93 |
| 938.79200000000003 | 0.969 | 0.93 |
| 940.70399999999995 | 0.965 | 0.929 |
| 942.61599999999999 | 0.964 | 0.93 |
| 944.52800000000002 | 0.966 | 0.931 |
| 946.44 | 0.967 | 0.932 |
| 948.35199999999998 | 0.969 | 0.934 |
| 950.26400000000001 | 0.97 | 0.935 |
| 952.17600000000004 | 0.971 | 0.937 |
| 954.08799999999997 | 0.971 | 0.938 |
| 956 | None | None |F/F0
(ms)
### Chart
| Category | |
|---|---|
| NC | 1.7826315789473686 |
| antagomir | 3.5699999999999994 |**
ΔF/F0
Antagomir
- +
PE
+ +
### Chart
| Category | |
|---|---|
| NC | 176.10044284539472 |
| antagomir | 140.10522539062498 |**
FDHM(ms)
Antagomir
- +
PE
+ +
PE+NC
PE+Antagomirs
E
### Chart
| Category | |
|---|---|
| C-NC | 1.0 |
| C-A | 0.6156385996852882 |
| ISO-NC | 1.2593098924921027 |
| ISO-A | 0.8843677741781076 |Myh7 mRNA Level
(Fold Change)
- + - +
Antagomirs
- - + +
ISO
*

## Slide 10
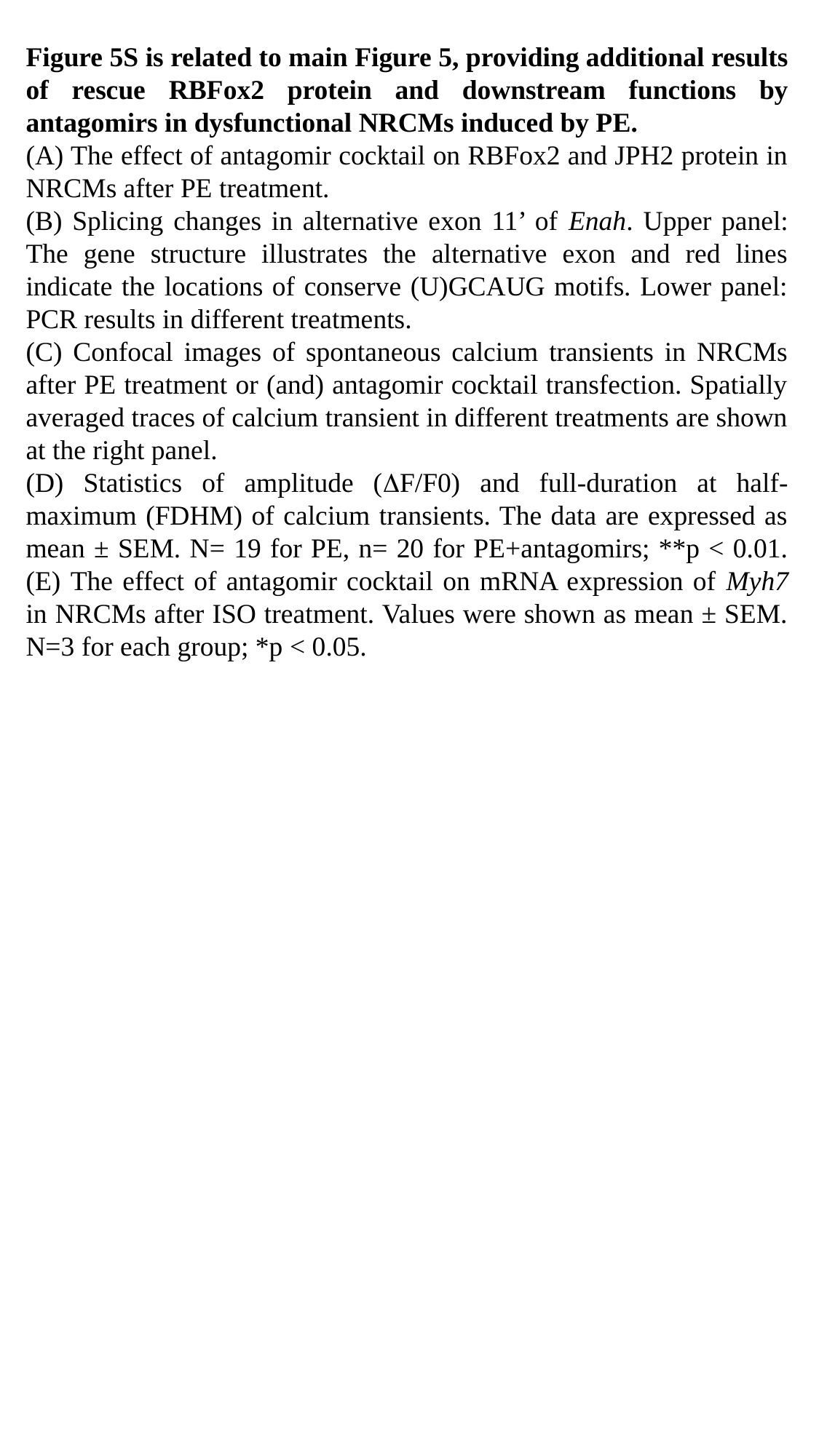

Figure 5S is related to main Figure 5, providing additional results of rescue RBFox2 protein and downstream functions by antagomirs in dysfunctional NRCMs induced by PE.
(A) The effect of antagomir cocktail on RBFox2 and JPH2 protein in NRCMs after PE treatment.
(B) Splicing changes in alternative exon 11’ of Enah. Upper panel: The gene structure illustrates the alternative exon and red lines indicate the locations of conserve (U)GCAUG motifs. Lower panel: PCR results in different treatments.
(C) Confocal images of spontaneous calcium transients in NRCMs after PE treatment or (and) antagomir cocktail transfection. Spatially averaged traces of calcium transient in different treatments are shown at the right panel.
(D) Statistics of amplitude (ΔF/F0) and full-duration at half-maximum (FDHM) of calcium transients. The data are expressed as mean ± SEM. N= 19 for PE, n= 20 for PE+antagomirs; **p < 0.01. (E) The effect of antagomir cocktail on mRNA expression of Myh7 in NRCMs after ISO treatment. Values were shown as mean ± SEM. N=3 for each group; *p < 0.05.
