# Supplementary material for: Suppression of RBFox2 by Multiple MiRNAs in Pressure Overload-Induced Heart Failure
Source: Int J Mol Sci. 2023 Jan 9;24(2):1283. doi: 10.3390/ijms24021283 (PMC9867119; doi:10.3390/ijms24021283)
Supplement: Supplementary file 1 [file ijms-24-01283-s001.zip › supplementary-table3.pdf]

Table S1. List of siRNA sequences

Gene name	sequence (5’ to 3’)
Rbfox2-Rat-652	sense：GGUAACCAGGAGCCAACAATT<br>antisense：UUGUUGGCUCCUGGUUACCTT
Rbfox2-Rat-960	sense：GCGACUACAUGUCUCUAAUTT<br>antisense：AUUAGAGACAUGUAGUCGCTT

Table S2. List of microRNA mimics sequences

Gene name	sequence (5’ to 3’)
mmu-miR-92a-3p mimics	UAUUGCACUUGUCCCGGCCUG<br>GGCCGGGACAAGUGCAAUAUU
mmu-let-7f-5p mimics	UGAGGUAGUAGAUUGUAUAGUU<br>CUAUACAAUCUACUACCUCAUU
mmu-miR-16-5p mimics	UAGCAGCACGUAAAUAUUGGCG<br>CCAAUAUUUACGUGCUGCUAUU
mmu-miR-200b-3p mimics	UAAUACUGCCUGGUAAUGAUGA<br>AUCAUUACCAGGCAGUAUUAUU
mmu-miR-24-3p mimics	UGGCUCAGUUCAGCAGGAACAG<br>GUUCCUGCUGAACUGAGCCAUU

Table S3. List of primer sequences

Gene name	Primer sequence (5’ to 3’)
mouse miR-1a-3p	TGGAATGTAAAGAAGTATGTAT
mouse miR-486-5p	TCCTGTACTGAGCTGCCCCGAG
mouse miR-128-3p	TCACAGTGAACCGGTCTCTTT
mouse miR-29c-3p	TAGCACCATTTGAAATCGGTTA
mouse miR-140-3p	TACCACAGGGTAGAACCACGG
mouse miR-30d-5p	TGTAAACATCCCCGACTGGAAG
mouse miR-342-3p	TCTCACACAGAAATCGCACCCGT
mouse miR-126a-3p	TCGTACCGTGAGTAATAATGCG
mouse miR-19b-3p	TGTGCAAATCCATGCAAAACTGA
mouse miR-126a-5p	CATTATTACTTTTGGTACGCG
mouse miR-30a-5p	TGTAAACATCCTCGACTGGAAG
mouse miR-10a-5p	TACCCTGTAGATCCGAATTTGTG
mouse miR-497a-5p	CAGCAGCACACTGTGGTTTGTA

mouse miR-29a-3p	TAGCACCATCTGAAATCGGTTA
mouse miR-24-3p	TGGCTCAGTTCAGCAGGAACAG
mouse miR-125b-5p	TCCCTGAGACCCTAACTTGTGA
mouse miR-23b-3p	ATCACATTGCCAGGGATTACC
mouse miR-126a-5p	TCGTACCGTGAGTAATAATGCG
mouse let-7f-5p	TGAGGTAGTAGATTGTATAGTT
mouse let-7a-5p	TGAGGTAGTAGGTTGTATAGTT
mouse snoRNA142	GTCAGTGCCACGTGTCTGG
mouse snoRNA135	CTAAAATAGCTGGAATTACCGGC
mouse pre-miR-125b	CTTGTGAGGTATTTTAGTAACATC
mouse pre-miR-200b	GCATTGGATAGTGTCTGATCTC
mouse pre-miR-140	GGTAGGTTACGTCATGCTGTTC
mouse pre-miR-24	TATCAGTTCTCATTTCACACAC
mouse pre-miR-16	CGTTAAGATTCTGAAATTACCT
mouse pre-miR-27b	ATTGGTGAACAGTGATTGGTTTC
mouse pre-miR-1a	GCCCATATGAACCTGCTAAGCTA
mouse pre-miR-92a	GGTGGCATTACTTGTGTTAGAT
mouse pre-let-7a	TAGCTCTCCAGCCATTGTGA
mouse pre-let-7c	CTACGCCAAGACTGACTG
mouse pre-let-7d	CTAGGAAGAGGTAGTAGGTTG
mouse pre-let-7e	CTGAGGTAGGAGGTTGTATAG
mouse pre-let-7f	GTGGGGTAGTGATTTTACCCTG
mouse pre-let-7g	TCCAGGCTGAGGTAGTAG
mouse pri-miR-125b	sense：GAGCCAGGATGTAGTCAC<br>antisense: CCTTCATTCTTTCTTGAGAC
mouse pri-miR-200b	sense：GACCTCTCCACTACCTATCT<br>antisense: CTCCGCCGTCATCATTAC
mouse pri-miR-140	sense：CTTGCTGGTGGTGTAGTC<br>antisense: TGTGGCTGTGTAGATGGA
mouse pri-miR-24	sense：GGAAACTGAGCCAACCTT<br>antisense: ACATGGAGACCTGGACTG
mouse pri-miR-16	sense：AGTAGCAGCACATAATGGTT<br>antisense: CTTACTTCAGCAGCACAGT
mouse pri-miR-27b	sense：CATTGCCAGGGATTACCA<br>antisense: GTTTCCAAAGAGCCACAAG
mouse pri-miR-1a	sense：TGAGGGAGGTGTAAGCAA<br>antisense: GCCTGTGTCTCAGTCTCT
mouse pri-miR-92a	sense：GCACATCTTCAGCATCCA<br>antisense: CCTCCTATTACACCAACCAA
mouse pri-let-7a	sense：CTGGATGTTCTCTTCACTGT<br>antisense: CCATTCCCATCATCTACTTGT
mouse pri-let-7c	sense：CCTCAAGAAGCCACAACA<br>antisense: ACTATCCAAGGTCTCTGTCA

mouse pri-let-7d	sense：CAAGTAGAAGACCAGCAAGA<br>antisense: CCACTTACGAGAACCACTG
mouse pri-let-7e	sense：CTGAGGTAGGAGGTTGTATAG<br>antisense: TCAAGATGGCATAGAGACTG
mouse pri-let-7f	sense：AGAGACTGATGCTTATTCCA<br>antisense: GCCTGGTCCTAGATACTTAC
mouse pri-let-7g	sense：CTTCCTCAAGTGCGTCCT<br>antisense: GAACAACTCCAAGCCTCTC
mouse RBFOX2	sense：GCTGTGTATGGTCCTGAGTTAT<br>antisense: GTGTTGATGCCTCCTCTTCC
mouse GAPDH	sense：TGACATCAAGAAGGTGGTGAAG<br>antisense: CCTGTTGCTGTAGCCGTATTC
mouse 18S	sense：GACAGGATTGACAGATTGATAG<br>antisense: CCAGAGTCTCGTTCGTTAT
rat RBFOX2	sense：CTCCTGACGCAATGGTTC<br>antisense: AGGTTATGTTCACTGGTCTG
rat GAPDH	sense：CCTGGTATGACAATGAATATGG<br>antisense: TCTTGCTCTCAGTATCCTTG
rat JPH2	sense：CACCACGGAGACCTACAT<br>antisense: GCGAACCTTGTTGCTCTT
rat 18S	sense：CTGAACCCGACTCCCTTT<br>antisense: GTGAACAGCAGTTGAACATG
rat Enah	sense：AGTAAGTCACCTGTCATCTCCA<br>antisense: TGCTTCAGCCTGTCATAGTCA
rat Sorbas2	sense：AACTGAAGACTTTAATAACACACC<br>antisense: CTGAATCTGGAGAGTGAGAC
rat Tlr4	sense：AATCGCATAGAGACATCCAA<br>antisense: ACAGCCAGCAATAAGTATCA
rat NPPA	sense：GTAGGATTGACAGGATTGGA<br>antisense: GTGATAGATGAAGACAGGAAG
rat NPPB	sense：CAGTCTCCAGAACAATCCA<br>antisense: GCTTGAACTATGTGCCATC

Table S4. List of antagomirs sequences

**antagomirs name**	**sequence (5’ to 3’)**
mmu-let-7a-5p	AACUAUACAACCUACUACCUCA
mmu-let-7b-5p	AACCACACAACCUACUACCUCA
mmu-let-7c-5p	AACCAUACAACCUACUACCUCA
mmu-let-7d-5p	AACUAUGCAACCUACUACCUCU
mmu-let-7e-5p	AACUAUACAACCUCCUACCUCA
mmu-let-7f-5p	AACUAUACAAUCUACUACCUCA
mmu-let-7g-5p	AACUGUACAAACUACUACCUCA
mmu-let-7i-5p	AACAGCACAAACUACUACUACCUCA
